# Neutralizing activity of Sputnik V vaccine sera against SARS-CoV-2 variants

**DOI:** 10.1038/s41467-021-24909-9

**Published:** 2021-07-26

**Authors:** Satoshi Ikegame, Mohammed N. A. Siddiquey, Chuan-Tien Hung, Griffin Haas, Luca Brambilla, Kasopefoluwa Y. Oguntuyo, Shreyas Kowdle, Hsin-Ping Chiu, Christian S. Stevens, Ariel Esteban Vilardo, Alexis Edelstein, Claudia Perandones, Jeremy P. Kamil, Benhur Lee

**Affiliations:** 1 Department of Microbiology at the Icahn School of Medicine at Mount Sinai, New York, NY USA; 2 Department of Microbiology and Immunology, Louisiana State University Health Shreveport, Shreveport, LA USA; 3 National Administration of Laboratories and Health Institutes of Argentina (ANLIS) Dr. Carlos G. Malbrán, Buenos Aires, Argentina

**Keywords:** Live attenuated vaccines, SARS-CoV-2, Viral immune evasion

## Abstract

Severe acute respiratory syndrome coronavirus 2 (SARS-CoV-2) has infected at least 180 million people since its identification as the cause of the current COVID-19 pandemic. The rapid pace of vaccine development has resulted in multiple vaccines already in use worldwide. The contemporaneous emergence of SARS-CoV-2 ‘variants of concern’ (VOC) across diverse geographic locales underscores the need to monitor the efficacy of vaccines being administered globally. All WHO designated VOC carry spike (S) polymorphisms thought to enable escape from neutralizing antibodies. Here, we characterize the neutralizing activity of post-Sputnik V vaccination sera against the ensemble of S mutations present in alpha (B.1.1.7) and beta (B.1.351) VOC. Using de novo generated replication-competent vesicular stomatitis virus expressing various SARS-CoV-2-S in place of VSV-G (rcVSV-CoV2-S), coupled with a clonal 293T-ACE2 + TMPRSS2 + cell line optimized for highly efficient S-mediated infection, we determine that only 1 out of 12 post-vaccination serum samples shows effective neutralization (IC_90_) of rcVSV-CoV2-S: B.1.351 at full serum strength. The same set of sera efficiently neutralize S from B.1.1.7 and exhibit only moderately reduced activity against S carrying the E484K substitution alone. Taken together, our data suggest that control of some emergent SARS-CoV-2 variants may benefit from updated vaccines.

## Abstract (web summary)

Here, the authors characterize the neutralization capacity of post-Sputnik V vaccination sera against SARS-CoV-2 variants of concern B.1.1.7 (alpha) and B.1.351 (beta), showing the latter to exhibit resistance to neutralization in vitro.

## Introduction

In the 15 months since its emergence in late 2019^1^, SARS-CoV-2 has caused over 131 million confirmed COVID-19 cases worldwide, leading to at least 2.85 million deaths^2^. SARS-CoV-2 is closely related to two other zoonotic betacoronaviruses, MERS-CoV and SARS-CoV, that also cause life-threatening respiratory infections^3^.

This global health emergency has spurred the development of COVID-19 preventive vaccines at an unprecedented pace. Six are already authorized for human use across the globe^4–9^. These vaccines focus on the SARS-CoV-2 spike protein (S), due to its critical roles in cell entry. Indeed, the presence of serum neutralizing antibodies directed at S correlate strongly with protection against COVID-19^10,11^. Although these six vaccines are efficacious, the recent emergence of SARS-CoV-2 variants has reignited concerns that the pandemic may not be so easily brought under control.

In December 2020, the United Kingdom reported the sudden emergence of a SARS-CoV-2 lineage, termed B.1.1.7 (501Y.V1, VOC 202012/01), which was designated as the first SARS-CoV-2 variant of concern (VOC). The lineage had rapidly increased in prevalence since first being detected in November 2020^12^. Its genome showed an unusually high number of non-synonymous substitutions and deletions, including eight in the S gene, suggesting a substantial degree of host adaptation that may have occurred during prolonged infection of an immunocompromised person^13^. The B.1.1.7 lineage has now been shown to exhibit enhanced transmissibility^14^ as well as an increased case fatality rate^15,16^.

Soon afterwards, two additional SARS-CoV-2 VOC, B.1.351 and P.1, were reported from S. Africa and Brazil, respectively, which each showed substantial escape from neutralizing antibodies elicited by first wave pandemic viruses, leading to documented cases of re-infection^17–19^. The S genes of B.1.351 and P.1 viruses each carry a number of mutations, but include three in the receptor binding domain (RBD) that are particularly notable, the S: N501Y substitution, found in B.1.1.7, alongside polymorphisms at positions 417 and 484, K417N/T and E484K. S: E484K had already been identified in multiple independent laboratories to confer escape from convalescent sera and monoclonal antibodies^20–22^. As expected, the P.1 and B.1.351 variants escape or resist neutralization by first wave convalescent sera, as well as antibodies elicited by COVID-19 vaccines^23–27^.

Although the P.1 and B.1.351 lineages are dominant in Brazil and S. Africa, unlike B.1.1.7 they have not increased greatly in number in the United States since originally being detected here. In contrast, the E484K polymorphism is recurrently emergent, and is found in a number of other lineages that are increasing in the U.S. and other countries. For example, a B.1.526 sub-lineage carrying E484K in recent weeks has expanded more rapidly than B.1.1.7^28,29^, which may be indicative of the ability of S: E484K variants to penetrate herd immunity. The P.2 lineage, originally detected in Rio de Janeiro, carries only the E484K mutation in the RBD and has spread to other parts of South America, including Argentina^30^.

The six COVID-19 vaccines currently in use around the world employ different strategies, and do not all incorporate the two proline substitutions that “lock” S into the pre-fusion conformer. Vaccines that do not utilize pre-fusion “locked” S are expected to produce lower levels of neutralizing antibodies, and hence may be less efficacious against infection, even if they do protect against severe COVID-19^31,32^. Indeed, a two-dose regimen of the AstraZeneca ChAdOx1 based vaccine, which does not use a “locked” S, did not protect against mild-to-moderate COVID-19 in S. Africa, where 93% of COVID-19 cases in trial participants were caused by the B.1.351 variant^33^. Like the AstraZeneca ChAdOx1 vaccine, the Sputnik V vaccine (Gam-COVID-Vac) is based on adenovirus vectored expression of a native S sequence, rather than a pre-fusion “locked” S^34^. Although the Sputnik V vaccine has a reported vaccine efficacy of 91.6% in the interim analysis of Phase 3 trials held in Russia between September 7 and November 24, 2020, none of the VOC mentioned above nor independent lineages containing the E484K mutation were prevalent in Russia during this time period. Since the Sputnik vaccine is now in use not only in Russia, but also in countries like Argentina, Mexico, and Hungary, where some of the VOC and emerging lineages bearing the E484K mutation are more widespread, it is critical to assess the neutralizing activity of Sputnik vaccine elicited antibody responses against these cognate VOC and mutant spikes.

This study characterizes the neutralization activity of sera from a dozen Sputnik V vaccine recipients in Argentina. Our work was spurred by Argentina’s nascent genomic surveillance efforts, which detected multiple independent lineages with S: E484K (B.1.1.318 and P.2) and/or S: N501Y substitutions (B.1.1.7 and P.1) in common, just as Argentina had started rolling out its vaccination campaign, which commenced on December 29, 2020. Here, we generated isogenic replication-competent vesicular stomatitis virus bearing the prevailing wild-type (WT = D614G) SARS-CoV-2 S (rcVSV-CoV2-S), or the B.1.1.7 (alpha variant), B.1.351 (beta variant) or E484K mutant S and used them in a robust virus neutralization assay. Our results show that Sputnik V vaccine sera effectively neutralized S: WT and S: B.1.1.7. viruses, albeit with highly variable titers. The same sera, however, exhibited moderate and markedly reduced neutralization titers, respectively, against S: E484K and S: B.1.351. Analyses of dose response curves indicate that S: B.1.351 exhibits resistance to neutralizing sera in a manner that is qualitatively different from the E484K mutant. Taken together, our data argue that surveillance of the neutralizing activity elicited by vaccine sera will be necessary on an ongoing basis. Viral neutralization assays can indicate which SARS-CoV-2 variants are likely capable of transmission in the face of vaccine elicited immunity, and whether updated vaccines will be needed to control their emergence and spread.

## Results

### Replication-competent VSV expressing SARS-CoV-2 spike proteins

Several groups have now generated replication-competent VSV expressing SARS-CoV-2 spike in place of VSV-G (rcVSV-CoV2-S)^35–38^. These rcVSV-CoV2-S can be used in BSL-2 compatible virus neutralization assays (VNAs), which correlate very well with VNAs using live SARS-CoV-2 (Spearman’s *r* > 0.9 across multiple studies). rcVSV-CoV2-S has been assessed as a candidate vaccine^37,39^, and used in forward genetics experiments to generate antibody escape mutants or perform comprehensive epitope mapping studies^20,38,40^. Indeed, the now concerning E484K mutation, present in many variants of concern (VOC), was identified as an antibody escape mutation using rcVSV-CoV-2-S^20,38^.

However, many groups passage their rcVSV-CoV-2-S extensively in Vero cells after the initial rescue, either to generate higher titer stocks and/or to remove confounding components such as the vaccinia virus expressing T7-polymerase and/or transfected VSV-G, both of which were deemed necessary for efficient rescue^38^. Serial passage of rcVSV-CoV-2-S in Vero cells invariably leads to mutations in the S1/S2 furin cleavage site, as well as truncations in the cytoplasmic tail of the S protein^39^. The latter promotes S incorporation into VSV without compromising the conformational integrity of the ectodomain, whereas the former is problematic when assessing the neutralization sensitivity and structure-function phenotype of Spike VOC with multiple mutations that likely have complex epistatic interactions.

**Fig. 1:**
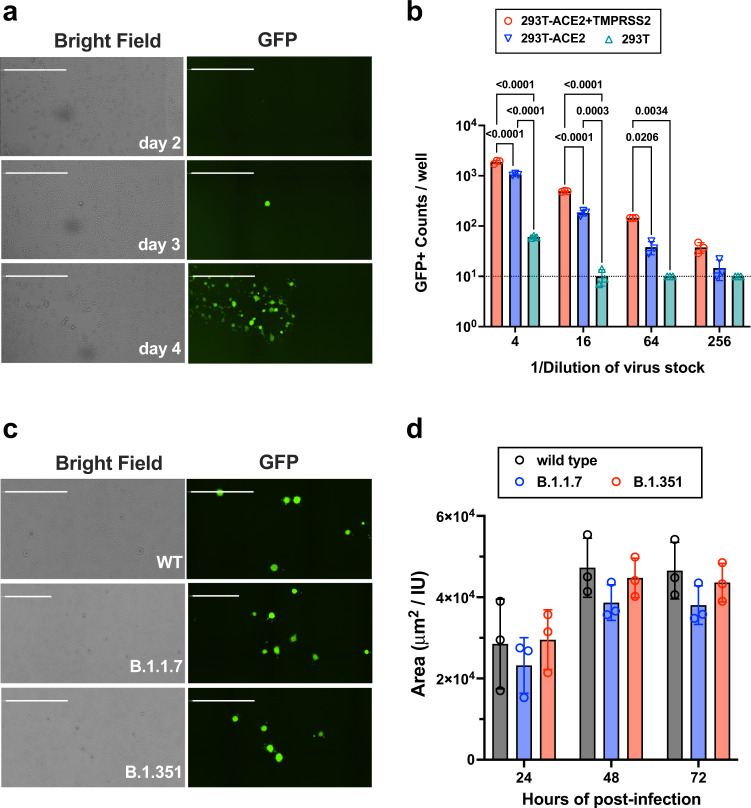
Replication-competent VSV bearing wild-type and variant SARS-CoV-2 spike (rcVSV-CoV2-S). **a** De novo generation of rcVSV-CoV2-S, carrying an EGFP reporter, in transfected 293T-ACE2 + TMPRSS2 (F8-2) cells as described in Supplementary Fig. 1. Single GFP + cells detected at 2–3 days post-transfection (dpt) form foci of syncytia by 4 dpt. Images taken by Celigo imaging cytometer (Nexcelom) and are computational composites from the identical number of fields in each well. White bar equals 1 millimeter. Foci is representative of at least 5 independent rescues. **b** Entry efficiency of rcVSV-CoV2-S in parental 293T, 293T-ACE2, or 293T-ACE2 + TMPRSS2 cells. Serial dilutions of virus stocks amplified on Vero-TMPRSS2 cells were used to infect the indicated cell lines in 96-well plates in triplicates. GFP + cells were detected and counted by the Celigo imaging cytometer at 10 h post-infection (hpi). Symbols are individual data points from triplicate infections at the indicated dilutions. Bars represent the average of three replicates with error bars indicating standard deviation. Adjusted *p*-values from a two-way ANOVA with Tukey’s multiple comparisons test are indicated in the graph. **c** rcVSV-CoV-2-S containing the prevailing WT (D614G) and VOC (B.1.1.7 and B.1.351) spikes were inoculated into one 6-well each of F8-2 cells (MOI 0.1) and subsequently overlaid with methylcellulose-DMEM to monitor syncytia formation. Representative images of syncytial plaques at 48 hpi are shown (out of three replicates per virus per timepoint). White bar equals 1 millimeter. **d** shows the growth of GFP + area per infectious unit (IU) in the six-well plate. GFP + areas were imaged and measured by the Celigo imaging cytometer at 24, 48, and 72 hpi. IU was checked at 10 hpi in the same well. GFP + area (μm^2^) at the indicated timepoint was divided by the IU counted at 10 hpi to normalize for input. Bar shows the average of triplicate experiments with error bar indicating standard deviation. Symbols represent individual data points. No statistically significant differences were detected between WT and VOC spikes in the size of GFP + syncytia at any given time point (two-way ANOVA as above, “ns” not indicated in graph). Source data are provided as a Source Data file.

To generate rcVSV-CoV2-S containing different variants or mutants on demand, without the need for extensive passaging, we developed a robust reverse genetics system and VNA which leverages the cell lines we previously developed for a standardized SARS-CoV-2 VNA that correlates well with live virus neutralization^41^. Salient improvements include the addition of a hammerhead ribozyme immediately upstream of the 3’ leader sequence, which cleaves in cis to give the exact 3’ termini, the use of a codon-optimized T7-polymerase which alleviates the use of vaccinia-driven T7-polymerase, and a highly permissive and transfectable 293T-ACE2 + TMPRSS2 clone (F8–2)^41^ (Supplementary Fig. 1). A 6-plasmid transfection into F8–2 cells resulted in GFP + cells 2–3 days post-transfection (dpt), which turned into foci of syncytia by 4–5 dpt indicating virus replication and cell-to-cell spread (Fig. 1a). Transfer of F8-2 cell supernatant onto interferon-defective Vero-TMPRSS2 cells allowed for rapid expansion of low-passage viral stocks that maintain only the engineered Spike mutations. Clarified viral supernatants from Vero-TMPRSS2 cells were aliquoted, sequenced verified, then titered on F8-2 cells to determine the linear range of response (Fig. 1b).

Next, we generated isogenic rcVSV-CoV2-S expressing the B.1.1.7, B.1.351, or E484K S to evaluate the neutralizing activity of Sputnik V vaccine sera from Argentina. Viruses bearing the alpha and beta variant spikes form spreading syncytia at a rate that was not significantly different from their wild-type counterpart (Fig. 1c, d).

### Neutralization activity of Sputnik vaccine sera

**Fig. 2:**
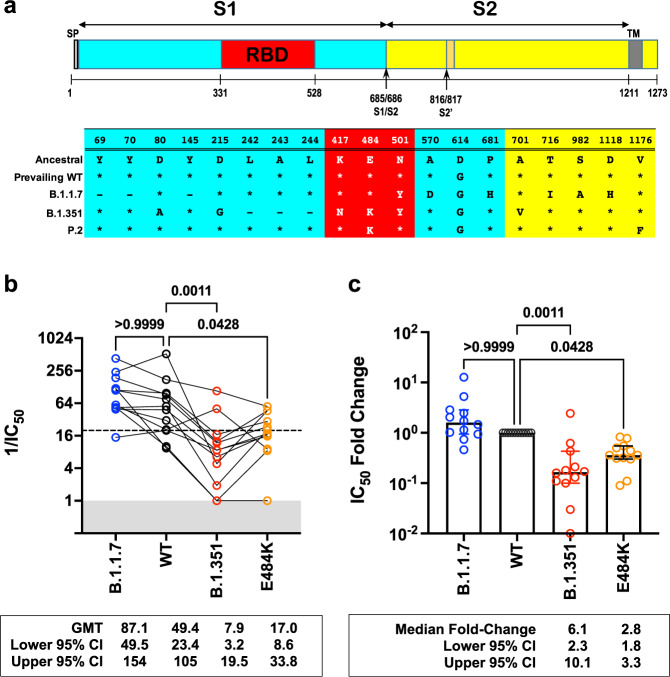
Neutralization activity of antibody responses elicited by the Sputnik V vaccine. **a** Schematic of the Spike substitutions that make up the variants being evaluated in this study. The amino acid positions and corresponding “Ancestral” sequence of the Wuhan isolate is shown. The prevailing WT sequence now has a D614G substitution. All the variants and mutants have D614G. (**b**) Neutralization activity of individual serum samples against rcVSV-CoV2-S with the WT (black circles), B.1.1.7 (blue circles), B.1.351 (red circles), or E484K (orange circles) spike proteins. Neutralization is represented by the reciprocal 50% inhibitory dilution factor (1/IC_50_). Sera samples with no appreciable neutralization against a given virus were assigned a defined 1/IC_50_ value of 1.0, as values ≤ 1 are not physiological (Gray shaded area). Dashed line indicates the lowest serum dilution tested (1/IC50 = 20). Geometric mean titers (GMT) and 95% CI for the neutralizing activity of all vaccine sera are indicated below each of the viral spike proteins examined. Adjusted *p*-values are indicated (non-parametric Friedman test with Dunn’s multiple comparisons test). **c** For each serum sample, the fold-change in IC_50_ (reciprocal inhibitory dilution factor) against the indicated variant and mutant spike proteins relative to its IC_50_ against wild-type (WT) spike (set at 1) is plotted. Adjusted *p*-values were calculated as in **b**. Medians are represented by the bars and whiskers demarcate the 95% CI. Neutralization dose–response curves were performed in triplicates, and the mean values from each triplicate experiment are shown as the single data points for each sera sample. Source data are provided as a Source Data file.

The relevant spike substitutions that make up these variants are indicated in Fig. 2a. The characteristics of the vaccine recipient cohort (*n* = 12) receiving the two-dose regimen of the Sputnik vaccine are given in Table 1. At one month post-completion of the two-dose regimen, the Sputnik V vaccine generated respectable virus neutralizing titers (VNT) against rcVSV-CoV2-S bearing the WT (D614G) and B.1.1.7 spike proteins (Fig. 2b). The geometric mean titer (GMT) and 95% CI for WT (1/IC_50_ GMT 49.4, 23.4-105) in our cohort of vaccine recipients was remarkably similar to that reported in the phase III Sputnik vaccine trial (GMT 44.5, 31.8-62.2)^42,43^. However, GMT against B.1.351 and E484K was reduced by a median 6.8- and 2.8-fold, respectively, compared to WT (Fig. 2c).

**Table 1:** Cohort characteristics of Sputnik vaccine recipients from ANLIS MALBRÁN (Buenos Aires, República Argentina).

Sera ID	First dose	Second dOSE	Vaccine status	Sex	Age
SP001	Late Dec/2020	Mid Jan/2021	(+)	M	45–50
SP002	Late Dec/2020	Mid Jan/2021	(+)	M	40–45
SP003	Late Dec/2020	Mid Jan/2021	(+)	M	55–60
SP004	Late Dec/2020	Mid Jan/2021	(+)	M	50–55
SP005	Late Dec/2020	Mid Jan/2021	(+)	M	35–40
SP006	Late Dec/2020	Mid Jan/2021	(+)	F	35–40
SP007	Late Dec/2020	Mid Jan/2021	(+)	F	20–25
SP008	Late Dec/2020	Early Feb/2021	(+)	M	35–40
SP009	Late Dec/2020	Early Feb/2021	(+)	F	30–35
SP010	Late Dec/2020	Mid Jan/2021	(+)	M	30–35
SP011	Late Dec/2020	Mid Jan/2021	(+)	M	40–45
SP012	Late Dec/2020	Mid Jan/2021	(+)	M	25–30
				Median age	39.5
				Range	25–56
SP013	N.A.	N.A.	(–)	F	45–50
SP014	N.A.	N.A.	(–)	F	50–55
SP015	N.A.	N.A.	(–)	M	40–45

*N.A*. not applicable

### Variable responses to SARS-CoV-2 variants

**Fig. 3:**
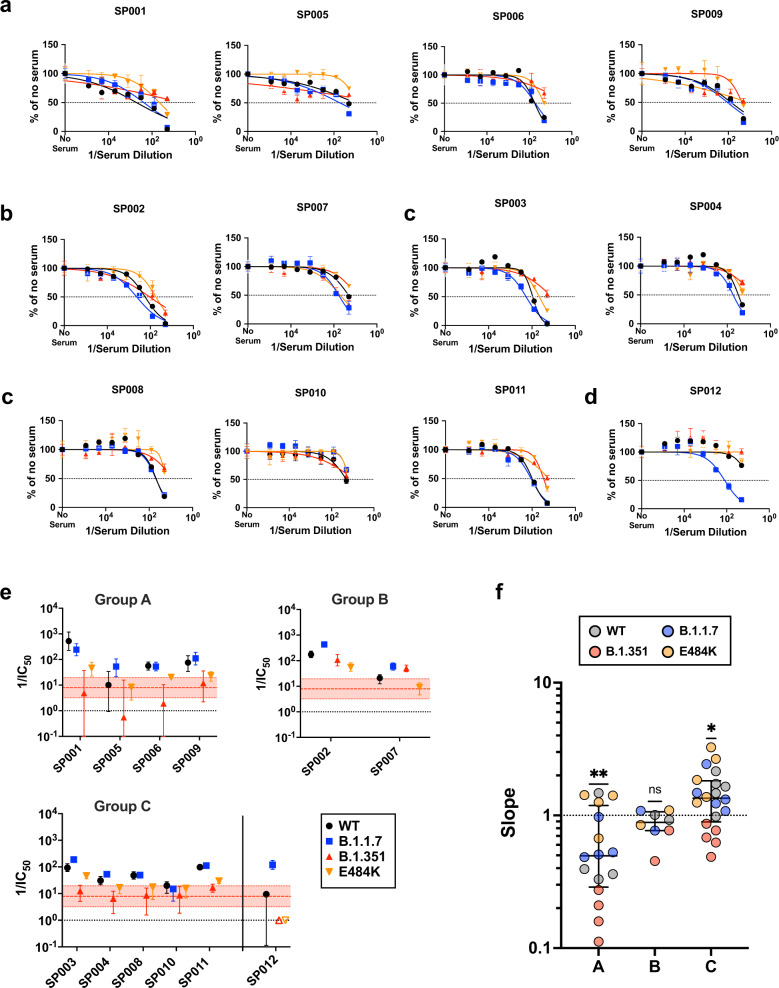
Sputnik vaccine recipients generate qualitatively different neutralizing antibody responses against SARS-CoV-2. **a**–**c** Group A (SP001, SP005, SP006, SP012), Group B (SP002, SP007), and Group C (SP003, SP004, SP008, SP010, SP011) represent potentially distinct classes of virus neutralizing activity present in the sera samples analyzed. Full neutralization curves for all sera tested against all viruses bearing the variant and mutant spike proteins are shown. **d** shows a singular example of a serum that only neutralized the B.1.1.7 spike. Data shown at each serum dilution point are normalized mean+/− standard deviation from triplicate infections. **e** graphs the serum neutralizing titers (SNT = 1/IC_50_) and 95% CI extrapolated from the nonlinear regression curves shown in **a**–**d** (*n* = 12). Colored filled symbols represent the indicated viruses, open symbols in **e** represent assigned SNT values of 1.0 when no significant neutralization activity could be detected (SP012: B.1.351 and E484K). The dotted black line represents a reciprocal serum dilution of 1.0. The red dashed line and shaded boundaries represent the geometric mean titer and 95% CI, respectively, for B.1.351 across all samples (*n* = 12). **f** The Hill slope values for all the neutralization curves are aggregated according to their groups. The different colored symbols in each group represent the indicated virus tested. The median (central bar) and interquartile range (whiskers) for each group are also shown. *p*-values (two-tailed) are from a non-parametric Wilcoxon-signed rank test comparing each group to a theoretical median of 1.0 (Group A, *n* = 16, Group B, *n* = 8, Group C, *n* = 20). ** = 0.0065, * = 0.0110, ns = 0.1953. Source data are provided as a Source Data file.

**Fig. 4:**
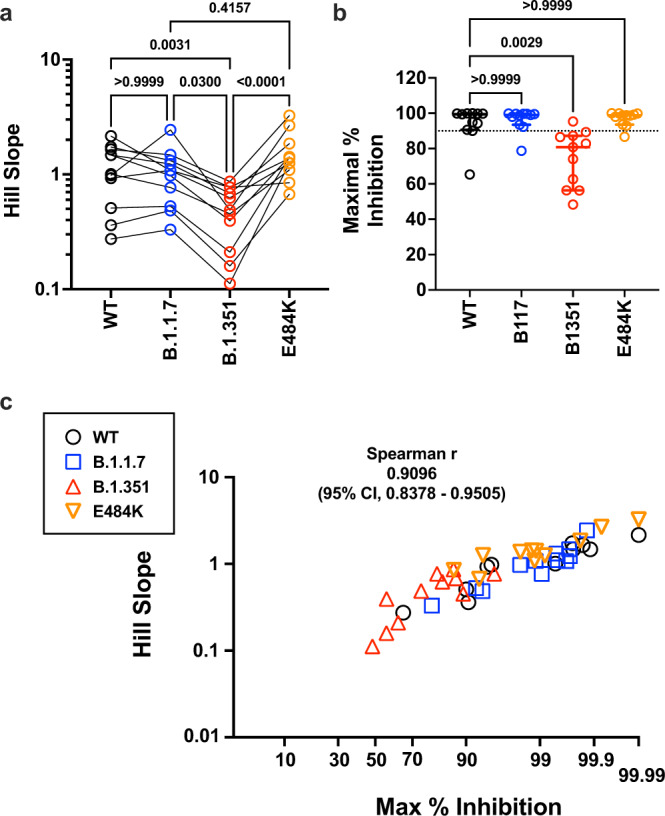
Maximal inhibition and slope help to define the distinct classes of neutralizing sera in Sputnik vaccine recipients. **a** Paired comparison of Hillslopes from the neutralization curves of all samples except for SP012 where no significant neutralization was observed for viruses other than B.1.1.7. Adjusted *p*-values are indicated (non-parametric Friedman test with Dunn’s multiple comparisons test, which assumes non-Gaussian distribution of values being analyzed). **b** Maximal percent inhibition (MPI) at full serum strength extrapolated from nonlinear regression of log(inhibitor) versus normalized response, variable slope curve. Model used is from PRISM v9.1.1 where ${y}=100/1+{10}^{({{\log }}{IC}50-{x})\times {Hillslope}}$. Log IC50 and Hill slope values were obtained for each curve generated in Fig. 3. MPI = 100 – *Y*, when *X* = 0 for reciprocal serum dilution of 1 (10^0^ = 1). Data points for one serum (SP012) against WT, B.1.351 and E484K could not be calculated because there was no best-fit value. The dotted line indicates 90% inhibition. Median (central bar) and interquartile values (whiskers) are indicated. Adjusted *p*-values was calculated as in **a**. Black, blue, red, and organe circles represent WT, B.1.1.7, B.1.351, and E484K viruses, respectively. **c** Correlation analysis of MPI versus the Hill Slope parameter for all sera samples tested against all spike proteins. SP012 was excluded for the abovementioned reasons. Non-parametric Spearman *r*-values and 95% confidence interval are shown. *x*-axis is plotted as an asymptotic cumulative probability scale as *x* approaches 100% (PRISM v9.1.1) only to resolve the many MPI values > 90%. Source data are provided as a Source Data file.

Sputnik vaccine recipients appeared to generate qualitatively different neutralizing antibody responses against SARS-CoV-2 that could be segregated into three different groups (Fig. 3). Group (A) sera showed reasonable VNT against wild-type (WT) and B.1.1.7 (Fig. 3a, e). However, the Hill slope of their neutralization curves for B.1.351 were relatively shallow (*h* < 0.40), resulting in a low IC50s and maximal neutralization of 50–60% even when extrapolated to full serum strength (Figs. 3e and  4). In contrast, Group (B) sera neutralized E484K and B.351 with similar potencies to WT and B.1.1.7, especially at high serum concentrations (Fig. 3b, e). This group of sera reveals that qualitatively different neutralizing responses can be generated that effectively neutralize B.1.351. Group (C) sera generally exhibited effective neutralization of WT, B.1.1.7, and even E484K at high serum concentrations, but not B.1.351 (Fig. 3c, e). The decreased potency and shallow Hill Slope resulted in <90% neutralization of B.1.351 even at full serum strength (see next section). One serum sample (SP012) exhibited little to no neutralizing activity against WT, E484K and B.1.351, yet it neutralized B.1.1.7 as well as Group A-C sera (Fig. 3d, e). That these three groups exhibit qualitatively distinct neutralization patterns is further highlighted by the Hill slopes of their neutralization curves (Fig. 3f). Group A sera not only have the lowest slopes against B.1.351, but as a group, they have slopes significantly <1 against the other viruses (median/IQR = 0.4965/0.2880–1.186). Group B sera mostly have slope values around 1 (median/IQR = 0.8855/0.7865–1.065) while Group C sera have the highest overall slopes (median/IQR = 1.348/0.8395–1.820) that are significantly >1 (Fig. 3f).

### Resistance to neutralization correlates with shallow Hill slopes

The Hill Slope of the neutralization curves against B.1.351 was significantly different from WT, B.1.1.7 and E484K (Fig. 4a). As a consequence, the maximal neutralization attainable when extrapolated to full serum strength was also significantly lower for B.1.351 compared to the rest (Fig. 4b). Conversely, the steep Hill slope for the E484K curves resulted in maximal neutralization potencies that were not significantly different from WT or B.1.1.7 despite significantly lower reciprocal IC_50_ values (compare Fig. 2b with Fig. 4b). Notably, the maximal percent inhibition was strongly correlated with the Hill Slope for WT and VOC/mutant spikes across all valid pairs of sample values (Fig. 4c, *n* = 45 pairs), suggesting that antibody co-operativity likely plays a role at high serum concentrations (see “Discussion”)^44^. While we acknowledge the limitations of extrapolating values from nonlinear regression curves, the striking correlation between slope and maximal percent inhibition attainable at full serum strength supports the robustness of our nonlinear regression model.

**Fig. 5:**
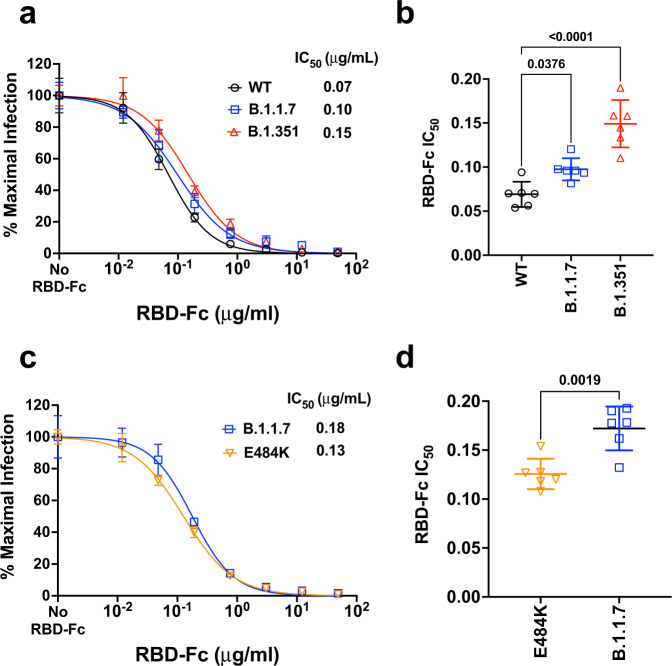
Competitive inhibition of rcVSV-CoV2-S entry by soluble RBD-Fc. **a** Recombinant RBD-Fc was serially titrated with the infection inoculum containing a fixed amount of rcVSV-CoV2-S bearing WT or the indicated VOC spike proteins. 10 hpi, GFP+ cells were quantified by the Celigo image cytometer. Data points are means of six replicates with error bars representing standard deviation. The number of GFP+ cells in the absence of any RBD-Fc was set to 100% and used to normalize the infection response in the presence of increasing amounts of RBD-Fc. Log[inhibitor] versus normalized response variable slope nonlinear regression curves were generated using GraphPad PRISM (v9.1.0). **b** The IC50 values from each replicate dose–response curve generated for a given virus were grouped. The mean (central bar) and standard deviation (whiskers) for each group are indicated. Adjusted *p*-values are indicated (ordinary one-way ANOVA with Dunnett’s multiple comparisons test are indicated). **c** is a repeat of the experiment done in **a** with the E484K mutant using a different preparation of recombinant RBD-Fc (see methods). B.1.1.7 serves as the common reference control. Data points are means of six replicates with error bars representing standard deviation. **d** The IC50 values were calculated as in **b**. The mean (central bar) and standard deviation (whiskers) for each group are indicated. Two-tailed *p*-value (0.0019) from an unpaired *t*-test is shown. Source data are provided as a Source Data file.

The heterogenous dose-response curves described in Figs. 3–4 is a property of Sputnik V vaccine elicited responses as soluble RBD-Fc inhibition of WT and VOC S-mediated entry produced classical dose response curves with Hill slopes close to −1.0 (Fig. 5). Both B.1.1.7 and B.1.351 were modestly but significantly more resistant to RBD-Fc inhibition (Fig. 5a, b). This is not surprising as both harbor the N501Y mutation known to enhance affinity of RBD for ACE2^45–47^. However, this 1.5 to 2-fold increase in RBD-Fc IC_50_ for B.1.1.7 and B.1.351, respectively, does not explain the neutralization-resistant versus sensitive phenotype of B.1.351 versus B.1.1.7 in our virus neutralization assays. Furthermore, the E484K mutant was more sensitive to RBD-Fc inhibition than B.1.1.7 (Fig. 5c, d), and yet also remained more neutralization-resistant relative to B.1.1.7. Experimental measurements of both RBD and trimeric spike binding to ACE2 have revealed that the E484K mutation alone does not confer increase binding affinity for ACE2 unlike N501Y^46,48^. Our RBD-Fc inhibition studies in the context of virus infection confirm and extend these results. Our data reinforces the notion that the mechanism underlying the increased neutralization resistance of E484K containing variants and mutants do not involve ACE2 binding affinity per se, but rather affects a key immunodominant epitope targeted by a significant class of human neutralizing antibodies, variably termed as RBM class II, RBS-B, or Cluster 2 antibodies^42,43,49,50^.

## Discussion

A key public health concern related to emergent SARS-CoV-2 variants is that by incrementally accruing mutations that escape neutralizing antibodies, they will penetrate herd immunity and spread to reach unvaccinated individuals, some of whom will be susceptible to severe or fatal disease.

Three of the six COVID-19 vaccines currently in use worldwide, namely Moderna mRNA-1273, BioNTech BNT162b2, and Janssen Ad26.COV2.S, each express S harboring K986P and V987P substitutions (2P) within a loop abutting the central helix of the S2′ membrane fusion machinery^51–53^. This modification locks the spike in a prefusion conformation and elicits higher titers of neutralizing antibodies^54,55^. The Janssen vaccine has an additional deletion in the furin cleavage site, while the yet-to-be approved Novavax vaccine contains arrays of stabilized spikes conjugated onto a nanoparticle (Table 2). Of the three vaccines that do not appear to make use of 2P Spike mutants, Gamaleya’s Sputnik V and AstraZeneca’s AZD1222 are adenovirus-vectored vaccines encoding native S. The third is CoronaVac, a preparation of inactivated SARS-CoV-2 virions. Although all six vaccines are highly efficacious at preventing severe COVID-19 outcomes, they do not all uniformly prevent infection. Moreover, in all cases thus far examined, these first generation vaccines are less effective against variants with certain non-synonymous substitutions in Spike, such as E484K.

**Table 2:** Summary of post-vaccine sera evaluated for neutralization potency against the indicated SARS-CoV-2 variants of concern (VOC).

			Neutralization assay (IC_50_ fold-reduction vs. WT)				
Vaccine	Company	Spike construct	B.1.1.7 (Alpha)	P.1 (Gamma)	B.1.351 (Beta)	Number of samples tested (*n*)	Reference (PMID)
Ad26.COV2.S	Johnson&Johnson	2P & ΔFurin	≤2× (n.s.)	NA	≤5×	8	33909009^a^
BNT162b2	Pfizer/BioNTech	2P	2×	NA	≤6.5×	10	33684923
BNT162b2	Pfizer/BioNTech	2P	2x (n.s.)	6.7×	35x	30	33743213
BNT162b2	Pfizer/BioNTech	2P	3.3x	NA	NA	25	33743891
BNT162b2	Pfizer/BioNTech	2P	NA	NA	7.9	25	33730597
mRNA-1273	Moderna	2P	1.8×	NA	≤8.6×	12	33684923
mRNA-1273	Moderna	2P	(n.s.)	4.5×	28x	35	33684923
BNT162b2 or mRNA-1273	Pfizer/BioNTech or Moderna	2P	NA	≤3×	NA	15^b^	33567448
NVX-CoV2373	Novavax	3Q – 2P	2×	NA	NA	28	33705729
AZD1222	AstraZeneca	Native	NA	NA	4×	13	33725432
AZD1222	AstraZeneca	Native	8.9x	NA	NA	49	33798499
AZD1222	AstraZeneca	Native	2.1–2.5x	NA	NA	25	33743891
AZD1222	AstraZeneca	Native	NA	NA	9x	25	33730597
CoronaVac	Sinovac	Native	NA	NA	NA	N.D.	N.D.
BBIBP-CorV	Sinopharm	Native	NA	NA	1.6× (n.s.)	12	33870240
				E484K			
Sputnik V	Gamaleya	Native	(n.s.)	2.8x (E484K)	6.1x	12	33821288^c^

^a^Non-human Primate data.

^b^Four are post-Pfizer/BioNTech and 11 are post-Moderna vaccine samples.

^c^Current study.

Table format adapted and updated from Abdool Karim and de Olivera^60^. *2P* 2 proline substitutions in the S2 central helix (K986P/V987P), Δ*Furin* deletion of furin cleavage site, *3Q–2P* RRAR to QQAQ to render furin cleavage site protease resistant combined with the aforementioned two proline substitutions.

The most concerning variants are those with multiple mutations in the receptor binding domain (RBD) that confer both enhanced affinity for the hACE2 receptor and escape from neutralizing antibody responses^17,24,27,33,56,57^. B.1.351 and P.1 have in common three RBD substitutions (K417N/T, E484K and N501Y) whereas B.1.351, P.1 and B.1.1.7 contain the N501Y substitution. Although B.1.1.7 shows enhanced transmissibility and more severe disease outcomes^52^, it does not appear to be consistently more resistant to serum neutralizing responses elicited by vaccines or natural infection^58,59^. The same is not true, however, for the B.1.351 variant.

In live virus plaque reduction neutralization assays, sera from AstraZeneca vaccine recipients in South Africa exhibited 4.1 to 32.5-fold reduction in neutralizing activity against B.1.351^33^. The actual reduction is even more marked because 7 of 12 vaccine recipients who had neutralizing activity against the parental B.1.1 variant, had undetectable neutralization against the B.1.351 strain. Comparator sera from recipients of Moderna and BioNTech mRNA vaccines showed smaller, 6.5- to 8.6-fold reductions in neutralization^60^.

Here, we showed that sera from Sputnik vaccine recipients in Argentina had a median 6.1-fold and 2.8-fold reduction in GMT against B.1.351 and the E484K mutant spike, respectively. Even more revealing is their dose-response curves. When extrapolated to full serum strength, half of the sera samples failed to achieve an IC_80_ and only 1 out 12 achieved an IC_90_ against B.1.351 (Fig. 4a). Table 2 summarizes peer-reviewed studies that have tested post-vaccination sera from the major vaccines against the VOC/mutant spikes used in this study. Our study shows a similar mean reduction in GMT (reciprocal IC50) against E484K and B.1.351 using 1-month post-Sputnik vaccine sera when compared to other vaccines. Our sample number is admittedly small but matches the median and modal number used in other studies to date. Nonetheless, we caution that comparing only the mean reduction in IC50 can be misleading as an aggregate measure of serum neutralizing activity. The neutralization curves for B.1.351 in our study are not classically sigmodal and have significantly shallower slopes than WT, B.1.17 and E484K, which result in ≤ 90% neutralization for all but one sample when extrapolated to full serum strength. The possible mechanisms for the varying slope values are discussed below.

E484K is present not only as part of an ensemble of RBD mutations present in B.1.351 and P.1, but in many of the 17 lineages detected from South America that carry it, such as P.2, E484K is the only RBD substitution (Supplementary Table 1). While the E484K substitution appears to be a common route of escape from many RBD-targeting monoclonal antibodies, it is somewhat surprising that a single mutation can confer a significant degree of neutralization resistance from polyclonal responses. Nonetheless, our data show that resistance conferred by E484K mutation be overcome by higher titer antibodies present in undiluted patient sera. But the neutralization resistance conferred by the suite of mutations present in B.1.351 appears qualitatively different. In the majority of cases, the slope of the dose response curve indicates a failure to neutralize even at full strength. We had previously shown that the dose-response curve slope is a major predictor of therapeutic potency for HIV broadly neutralizing antibodies at clinically relevant concentrations^44^. Importantly, the slope parameter is independent of IC50 but is specifically related to an antibody’s epitope class. Here, we show that defining the neutralization phenotype of a given spike variant or mutant by both its relative IC50 and slope provides a fuller characterization of serum neutralizing activity against SARS-CoV-2 and emergent VOC.

The deletion of residue 242–244 in the NTD of the B.1.351 spike appear to cause large-scale resurfacing of the NTD antigenic surface resulting in greater conformational heterogeneity^46^. Variable neutralization responses across such a heterogenous virus population may result in the shallow slopes (<1) seen. Furthermore, three major classes of neutralizing antibodies (RBS-A, -B, and -C) identified from convalescent patients are sensitive to either the K417N (RBS-A) or E484K (RBS-B and -C) substitutions present in B.1.351^61^. On the other hand, at high serum concentrations, co-operative effects from low-affinity spike binding antibodies or increased spike occupancy by different classes of antibodies can result in the steep Hill slope observed. The steep Hill slopes (>1.0) observed for E484K suggest such co-operative effects might be occurring.

The emergence of variants is fluid situation. B.1.427/1.429, B.1.526, and B.1.617 are other emergent VOI/VOC that could be tested. These strains have substitutions in the RBD (L452R and E484K/Q) and elsewhere in the spike that might confer some degree of neutralization resistance in our in vitro assays. However, all vaccines are effective against most variants, and we do not know what degree of resistance in in vitro assays translate to a decrease in the real world efficacy of any given vaccine.

Although we stress that the Gameyla Sputnik V vaccine is likely to retain strong efficacy at preventing severe COVID-19, even in the case of infection by VOC, our data reveal a concerning potential of B.1.351, and to a lesser extent, any variant carrying the E484K substitution (e.g. P.2), to escape the neutralizing antibody responses that this immunization elicits. Furthermore, we acknowledge that in vivo protective efficacy can be derived from Fc effector functions of antibodies that bind but do not neutralize. In addition, an adenoviral vectored vaccine should induce potent cell-mediated immunity against multiple epitopes, which were not measured in our study. Nevertheless, given the crucial roles neutralizing antibodies play in preventing infection, our results suggest that updated SARS-CoV-2 vaccines may be necessary to eliminate the virus.

## Methods

### Cell lines

Vero-CCL81-TMPRSS2 (ATCC CCL-81 derived), HEK 293T-hACE2 (clone 5–7), and 293T-hACE2-TMPRSS2 (clone F8-2) cells (ATCC CRL-3216 derived) were described previously^41^, and were maintained in Dulbecco’s modified Eagle’s medium (DMEM, Gibco) containing 10% fetal bovine serum (FBS, R&D Systems). The HEK 293T-hACE2-TMPRSS2 cells were plated on collagen coated plates or dishes. BSR-T7/5 cells^62^(RRID: CVCL_RW96), which stably express T7-polymerase were maintained in DMEM with 10% FBS.

### VSV-eGFP-CoV2 spike (∆21aa) genomic clone and helper plasmids

We cloned VSV-eGFP sequence into pEMC vector (pEMC-VSV-eGFP), which includes an optimized T7 promoter and hammerhead ribozyme just before the 5’ end of the viral genome. The original VSV-eGFP sequence was from pVSV-eGFP, a generous gift of Dr. John Rose^63^.

We generated pEMC-VSV-eGFP-CoV2-S (Genbank Accession: MW816496) as follows: the VSV-G open reading frame of pEMC-VSV-eGFP was replaced with the SARS-CoV-2 S, truncated to lack the final 21 amino acids^64^. We introduced a PacI restriction enzyme site just after the open reading frame of S transcriptional unit, such that the S transcriptional unit is flanked by MluI/PacI sites. SARS-CoV-2 S is from pCAGGS-CoV-2-S^65^, which codes the codon optimized S from the Wuhan Hu-1 isolate (NCBI ref. seq. NC_045512.2) with a point mutation of D614G, resulting in B.1 lineage. The B.1.1.7 Spike we used carries the mutations found in GISAID Accession Number EPI_ISL 668152: del 69–70, del145, N501Y, A570D, D614G, P681H, T716I, S982A, and D1118H. The B.1.351 Spike carries the mutations D80A, D215G, del242-244, K417N, E484K, N501Y, D614G, and A701V (from EPI_ISL_745109). The Spike sequences of WT, B.1.1.7, B.1.351, and E484K are available at Genbank (Accession Numbers: MW816497, MW816498, MW816499, and MW816500; please also see Supplemental Table 2).

Sequences encoding the VSV N, P, M, G, and L proteins were also cloned into pCI vector to make expression plasmids for virus rescue, resulting in plasmids: pCI-VSV-N, pCI-VSV-P, pCI-VSV-M, pCI-VSV-G, and pCI-VSV-L. These accessory plasmids were a kind gift from Dr. Benjamin tenOever.

### Generation of VSV-CoV2 spike from cDNA

In all, 4 × 10^5^ 293T-ACE2-TMPRSS2 cells per well were seeded onto collagen-I coated 6-well plates. The next day, 2000 ng of pEMC-VSV-EGFP-CoV2 spike, 2500 ng of pCAGGS-T7opt^66^, 850 ng of pCI-VSV-N, 400 ng of pCI-VSV-P, 100 ng of pCI-VSV-M, 100 ng of pCI-VSV-G, 100 ng of pCI-VSV-L were mixed with 5.5 μL of Plus reagent and 8.9 μL of Lipofectamine LTX (Invitrogen). Thirty minutes later, transfection mixture was applied to 293T-hACE2-TMPRSS2 cells in a dropwise fashion. Medium was replaced with DMEM-10% FBS at 24 h post-transfection and cells maintained for 4 to 5 days until GFP positive syncytia appeared. Rescued viruses were amplified in Vero-CCL81-TMPRSS2 cells^41^, then titrated on 293T-ACE2-TMPRSS2 cells and used for the virus neutralization assay.

### Virus neutralization assay

In all, 5 × 10^4^ 293T-hACE2-TMPRSS2 cells per well were seeded onto collagen-coated 96-well cluster plates one day prior to use in viral neutralization assays. Virus stocks were pre-mixed with 4-fold serially diluted serum (1:20 to 1:81,920) in DMEM 10%FBS for 10 min at room temperature before transferring the virus plus serum mix onto target cells. Note: all sera assayed in this study were previously heat inactivated (56 °C for 30 min) before use in any viral neutralization studies. At 10 h post-infection, GFP counts were acquired by the Celigo imaging cytometer (Nexcelom Biosciences, version 4.1.3.0). Each assay was done in triplicate. For calculation of IC_50_, GFP counts from “no serum” conditions were set to 100%; GFP counts of each condition (serum treated) were normalized to no serum control well. Inhibition curves were generated using Prism 8.4.3 (GraphPad Software) with “log (inhibitor) vs. normalized response-variable slope” settings.

### Design of RBD-Fc producing Sendai virus

Sendai virus (SeV) Z strain cDNA sequence (AB855655.1) was generated and cloned into pRS vector with the addition of eGFP transcriptional unit at the head of SeV genome. The sequence of F transcriptional unit was from SeV fushimi strain (KY295909.1) due to the cloning reason. We refer to the pRS-based plasmid coding this sequence as pRS-SeVZ-GFP-F^fushimi^ in this paper. For the introduction of foreign gene into SeV, we generated additional transcriptional unit for RBD-Fc between P gene and M gene. RBD-Fc construct was generated as below; codon optimized DNA sequence of from SARS-CoV-2 spike (MN908947) in pCAGGS a gift of Dr. Florian Krammer^65^. S amino acids 319–541 (corresponding to the RBD domain) sequence were C-terminally fused to the Fc region of human IgG_1_ (220–449 aa of P0DOX5.2)

### Generation of recombinant Sendai virus from cDNA

In all, 2 × 10^5^ BSR-T7 cells per well were seeded onto 6-well cluster plates. The next day, 4 µg of pRS-SeVZ-GFP-F^fushimi^, 4 µg of pCAGGS-T7opt, 1.44 µg of SeV-N, 0.77 ug of SeV-P, 0.07 ug of SeV-L were mixed with 5.5 μL of Plus reagent and 8.9 μL of Lipofectamine LTX (Invitrogen). 30 min later, transfection mixtures were applied to Bsr-T7 cells in a dropwise fashion, as described previously^66^. At one day post transfection, medium was replaced with DMEM + 0.2 µg/mL of TPCK-trypsin (Millipore Sigma, #T1426), with subsequent medium replacement each day until infection reached 100% cytopathic effect. Supernatants were stored at −80 °C until use in experiments.

### Titration of viruses

For SeV titration, 2 × 10^4^ Bsr-T7 cells per well were seeded onto 96-well plates. The next day, 100 µL of serially diluted virus stock (in DMEM-10% FBS) were applied to each well. GFP positive foci were counted at 24 h post-infection using a Celigo imaging cytometer (Nexcelom, Inc.). Infectivity is presented in infectious units (IU) per mL.

For VSV-CoV2 titration, 5 × 10^4^ 293T-hACE2-TMPRSS2 cells per well were seeded onto a collagen-coated 96-well plate. Serially diluted virus stocks were then applied to the cells, and GFP positivity was scored at 10 h post-infection using a Celigo imaging cytometer.

### Production of proteins and purification

In all, 5 × 10^6^ Bsr-T7 cells are seeded in T175cm^2^-flask one day before infection. Cells were infected by SeV at MOI of 0.1 for one hour, followed by replacement of medium with DMEM supplemented with 0.2 mg/mL TPCK-trypsin. Medium was replaced with fresh 0.2 mg/mL TPCK-trypsin containing DMEM each day until infection reached 100% cytopathic effect, at which point medium was exchanged for DMEM lacking TPCK-trypsin. Cells were incubated for additional 24 h to allow protein production. Supernatants were centrifuged at 400 x *g* for 10 min at 4 °C to remove cellular debris, then passed through a 0.1 µm filter (Corning^®^, 431475) to remove virions, and made up to a final 0.1%TX-100 (Millipore Sigma, T8787) to inactivate any remaining virions. Supernatant containing RBD-Fc was applied to Protein G Sepharose (Millipore Sigma, GE17-0618-01) containing column (10 mL Polypropylene Columns; ThermoFisher, 29922), followed by 3X PBS wash and elution with 9 mL of 0.1 M Glycine buffer (pH 2.7) directly into 1 mL of 2 M Tris-HCl (pH 10) neutralization buffer. Purified RBD-Fc was buffer exchanged 3X with PBS and concentrated in an Amicon Ultra-15 centrifugal filter (50 kDa cutoff, UFC905024). Protein concentration was determined by Bradford assay.

### Serum collection

Sera were collected from Sputnik V vaccinated individuals recruited for this study by investigators at the Administración Nacional de Laboratorios e Institutos de Salud “Dr. Carlos Malbrán” (ANLIS MALBRAN) in Buenos Aires, Argentina (A.E.V, A.E, C.P.). See Human Subjects Research below. Volunteers received their first dose either on 29 December or 31 December 2020, and their second dose either on 20 January 2021 or 2 February 2021. Other patient characteristics are detailed in Table 1. Data presented in this study were from sera collected on 22 February 2021 via venipuncture directly into serum separator tubes (BD Vacutainer SST™ tubes). Serum was extracted after centrifugation as per manufacturer’s instructions and was couriered the same day on dry ice to the Icahn School of Medicine at Mount Sinai (New York, USA). At least 6 mL of de-identified serum from each sample was received in New York and remained frozen until analysis was performed.

### Human subjects research

Human subjects research was conducted following the Declaration of Helsinki and related institutional and local regulations. Studies and serum collection relating to the Sputnik vaccine at ANLIS Dr. Carlos G. Malbrán (National Administration Laboratories and Health Institutes—Carlos G. Malbrán, Argentina) were approved by the Research Ethics Committee of its Unidad Operativa Centro de Contención Biológica (UOCCB) on 9 February 2021. Written informed consent was obtained as per institutional policy.

## Supplementary information

Supplementary Information

Peer Review File

Updated nr-reporting-summary

## Data Availability

Source data are provided with this paper. Raw images of fluorescent microscopy data presented in Fig. 1a, c are deposited in Figshare and can be accessed using 10.6084/m9.figshare.14919732 and 10.6084/m9.figshare.14916627, respectively. The spike sequences of WT, B.1.1.7, B.1.351, and E484K used to generate our rcVSV-CoV- 2-S are available at Genbank (Accession Numbers: MW816497, MW816498, MW816499, and MW816500; Supplementary Table 2). Cell lines and viruses can be obtained under a materials transfer agreement from the corresponding author (benhur.lee@mssm.edu). Source data are provided with this paper.

## Source data

Source Data
