# Supplementary material for: Neutralizing activity of Sputnik V vaccine sera against SARS-CoV-2 variants
Source: Nat Commun. 2021 Jul 26;12:4598. doi: 10.1038/s41467-021-24909-9 (PMC8313705; doi:10.1038/s41467-021-24909-9)
Supplement: Supplementary file 3 — Updated nr-reporting-summary [file 41467_2021_24909_MOESM3_ESM.pdf]

nature portfolio

Corresponding author(s): Benhur Lee
Last updated by author(s): Jul 8, 2021

Reporting Summary

Nature Portfolio wishes to improve the reproducibility of the work that we publish. This form provides structure for consistency and transparency in reporting. For further information on Nature Portfolio policies, see our Editorial Policies and the Editorial Policy Checklist.

Statistics

For all statistical analyses, confirm that the following items are present in the figure legend, table legend, main text, or Methods section.

n/a	Confirmed	
☐	☒	The exact sample size (*n*) for each experimental group/condition, given as a discrete number and unit of measurement
☐	☒	A statement on whether measurements were taken from distinct samples or whether the same sample was measured repeatedly
☐	☒	The statistical test(s) used AND whether they are one- or two-sided<br>*Only common tests should be described solely by name; describe more complex techniques in the Methods section.*
☒	☐	A description of all covariates tested
☐	☒	A description of any assumptions or corrections, such as tests of normality and adjustment for multiple comparisons
☐	☒	A full description of the statistical parameters including central tendency (e.g. means) or other basic estimates (e.g. regression coefficient) AND variation (e.g. standard deviation) or associated estimates of uncertainty (e.g. confidence intervals)
☐	☒	For null hypothesis testing, the test statistic (e.g. *F*, *t*, *r*) with confidence intervals, effect sizes, degrees of freedom and *P* value noted<br>*Give P values as exact values whenever suitable.*
☒	☐	For Bayesian analysis, information on the choice of priors and Markov chain Monte Carlo settings
☒	☐	For hierarchical and complex designs, identification of the appropriate level for tests and full reporting of outcomes
☒	☐	Estimates of effect sizes (e.g. Cohen's *d*, Pearson's *r*), indicating how they were calculated

Our web collection on statistics for biologists contains articles on many of the points above.

Software and code

Policy information about availability of computer code

Data collection	Celigo (Nexcelom Biosciences, version 4.1.3.0) mentioned in Methods
Data analysis	GraphPad PRISM v8.3.4, v9.1.0, v9.1.1

For manuscripts utilizing custom algorithms or software that are central to the research but not yet described in published literature, software must be made available to editors and reviewers. We strongly encourage code deposition in a community repository (e.g. GitHub). See the Nature Portfolio guidelines for submitting code & software for further information.

Data

Policy information about availability of data

All manuscripts must include a data availability statement. This statement should provide the following information, where applicable:

- Accession codes, unique identifiers, or web links for publicly available datasets
- A description of any restrictions on data availability
- For clinical datasets or third party data, please ensure that the statement adheres to our policy

Source data are provided with this paper. Raw images of fluorescent microscopy data presented in Fig. 1a and 1c are deposited in Figshare and can be accessed using DOI: 10.6084/m9.figshare.14919732 and 10.6084/m9.figshare.14916627, respectively. The spike sequences of WT, B.1.1.7, B.1.351, and E484K used to generate our rcVSV-CoV- 2-S are available at Genbank (Accession Numbers: MW816497, MW816498, MW816499, and MW816500; Supplementary Table 2). Cell lines and viruses can be obtained under a materials transfer agreement from the corresponding author (benhur.lee@mssm.edu).

Field-specific reporting

Please select the one below that is the best fit for your research. If you are not sure, read the appropriate sections before making your selection.

- [x] Life sciences
- [ ] Behavioural & social sciences
- [ ] Ecological, evolutionary & environmental sciences

For a reference copy of the document with all sections, see nature.com/documents/nr-reporting-summary-flat.pdf

Life sciences study design

All studies must disclose on these points even when the disclosure is negative.

Sample size	No sample size calculations were performed. Vaccine sera was obtained as per recruitment availability subject to IRB, time constraints, and public health urgency.
Data exclusions	No data were excluded.
Replication	All attempts at replication were successful. The number of replicates (n= 3 to 6) for all experiments are indicated in the figure legends.
Randomization	Not relevant. The first 12 sera samples recruited from post-vaccination volunteers were all analyzed. Vaccine sera were obtained as per recruitment availability subject to IRB, time constraints and public health urgency.
Blinding	Vaccine cohort recipients are known to the investigators collecting the samples. But samples were sent blinded for analysis of neutralizing activity. Vaccine recipient data in Table 1 was filled in just prior to final submission after all data analysis have been performed.

Reporting for specific materials, systems and methods

We require information from authors about some types of materials, experimental systems and methods used in many studies. Here, indicate whether each material, system or method listed is relevant to your study. If you are not sure if a list item applies to your research, read the appropriate section before selecting a response.

Materials & experimental systems

n/a	Involved in the study	
x		Antibodies
	x	Eukaryotic cell lines
x		Palaeontology and archaeology
x		Animals and other organisms
	x	Human research participants
x		Clinical data
x		Dual use research of concern

Methods

n/a	Involved in the study	
x		ChIP-seq
x		Flow cytometry
x		MRI-based neuroimaging

Eukaryotic cell lines

Policy information about cell lines

Cell line source(s)	293T-ACE2+TMPRSS2 (clone F8-2), 293T-ACE2 (clone 5-7), Vero-CCL81-TMPRSS2 were previously established and characterized in the corresponding author's lab (B.L.) at the Icahn School of Medicine (PMID:33593976). ATCC numbers are provided for the relevant parental cells (Vero-CCL81, HEK 293T) in METHODS. Parental HEK-293T cells used in Fig. 1b.
Authentication	Parental 293T authenticated via ATCC STRB5027 (Test date: 01-18-2021; 100% matched to CRL-3216 293T Cells; Embryonic Kidney Cells, Human). F8-2 (293T-ACE2+TMPRSS2) and F5-7 (293T-ACE2) clones and Vero-CCL81-TMPRSS2 cells authenticated via RT-qPCR fpr ACE2 and/or TMPRSS2 as described in PMID:33593976.
Mycoplasma contamination	All cell lines are confirmed to be mycoplasma negative using MycoAlert mycoplasma detection kit (Lonza).
Commonly misidentified lines (See ICLAC register)	No commonly misidentified cell line was used in this study.

Human research participants

Policy information about studies involving human research participants

Population characteristics	Described in Table 1 of the manuscript.
Recruitment	Volunteers. Recruitment as per procedure approved by Research Ethics Committee of ANLIS-MALBRAN (Srgentina). The first 12 sera samples recruited from post-vaccination volunteers were all analyzed. Vaccine sera were obtained as per recruitment availability subject to IRB, time constraints and public health urgency. Vaccinees are in the priority cohort under Argentinean Health Ministry guidelines, which includes front-line healthcare workers.
Ethics oversight	Studies and serum collection relating to the Sputnik vaccine at ANUS Dr. Carlos G. Mal bran (National Administration Laboratories and Health Institutes - Carlos G. Mal bran, Argentina) were approved by the Research Ethics Committee of its Unidad Operativa Centro de Contencion Biologica (UOCCB) on 9 Feb 2021. Written informed consent was obtained. Details are provided in Human Subjects Research Section. IRB is on file with the corresponding author and at ANLIS-MALBRAN (Argentina).

Note that full information on the approval of the study protocol must also be provided in the manuscript.
