# Supplementary material for: Neutralizing activity of Sputnik V vaccine sera against SARS-CoV-2 variants
Source: Nat Commun. 2021 Jul 26;12:4598. doi: 10.1038/s41467-021-24909-9 (PMC8313705; doi:10.1038/s41467-021-24909-9)
Supplement: Supplementary file 2 — Peer Review File [file 41467_2021_24909_MOESM2_ESM.pdf]

Editorial Note: This manuscript has been previously reviewed at another journal that is not operating a transparent peer review scheme. This document only contains reviewer comments and rebuttal letters for versions considered at *Nature Communications*.

Reviewers' Comments:

Reviewer #1:
Remarks to the Author:
Although the authors have only verbally addressed most of this reviewer’s comments, due to time sensitivity of the topic, this reviewer supports the publication of the study in Nature Communications.

Reviewer #3:
Remarks to the Author:
The authors have now substantially revised their manuscript with respect to the presentation and interpretation of the data. By now it is an excellent scientific report on the efficacy of the Sputnik vaccine against SARS-CoV-2 and its VOC. Appropriate statistics are now used and the Hill-coefficients presented and discussed as relevant parameters of "broadness" of the humoral response. This presents a solid basis to compare efficacy of the Sputnik vaccine to that of other vaccines. In view of the widespread use of the vaccine, this is of "global" interest to a broad readership.

As a side kick, the authors also present a new version of the pseudotyped virus neutralization assay used to determine the ability of antibodies to block infection of target cells.
